# Understanding intravaginal practices for gynecologic health among Haitian migrant women: A mixed-methods analysis

**DOI:** 10.1371/journal.pgph.0007394

**Published:** 2026-10-29

**Authors:** Dominique Guillaume, Laetitia Mélissande Amédée, Maria Alcaide, Joy Chepkorir, Ashley Gresh, Ann Augustin, Laeticia Jean Baptiste, Kamila Alexander

**Affiliations:** 1 Rory Meyers College of Nursing, New York University, New York, New York, United States of America; 2 Department of Psychiatry & Behavioral Neurosciences, McMaster University, Hamilton, Canada; 3 Miller School of Medicine, University of Miami, Miami, Florida, United States of America; 4 School of Nursing, Johns Hopkins University, Baltimore, Maryland, United States of America; 5 Department of Education and Human Development, University of Miami, Miami, Florida, United States of America; 6 School of Social Work, University of Maryland, Baltimore, Maryland, United States of America; Worcester Polytechnic Institute, UNITED STATES OF AMERICA

## Abstract

Haitian migrant women in the U.S. experience disproportionate rates of cervical cancer. Intravaginal practices (IVPs) are widely used among Haitian women and have been linked to an increased risk of human papillomavirus (HPV) infection. Although IVPs are well-documented in the Haitian community, little is known about Haitian migrant women’s perceptions and motivations underlying their use. This exploratory sequential mixed-methods study examined Haitian migrant women’s motivations for engaging in IVPs, products used for IVPs, and indications for their use. A convenience sample of Haitian migrant women in South Florida were recruited through active and passive recruitment approaches in community-based centers. Semi-structured qualitative interviews (n = 25) explored perceptions, motivations, and products used for IVPs. Qualitative results informed the development of survey items measuring IVPs, which were administered to participants (n = 151). Chi-square analysis with a Bonferroni adjustment examined associations between IVP products and IVP indications. All women in the qualitative sample (n = 25, 100%) confirmed IVP use compared to 68.9% (n = 104) of women in the quantitative sample. Qualitative interviews revealed that IVP preferences were rooted in cultural expectations with most women starting these practices either during puberty or after sexual debut. IVP products (e.g., plant and herbal agents, astringent washes, and antibiotics) were often chosen based on specific gynecologic indications, with this finding being consistent across both samples. Pigeon pea (*pwa kongo*) was the most frequently used IVP product in the quantitative sample (n = 74, 71.2%) and was associated with nearly all IVP indications (p ≤ 0.004). Given the high prevalence of IVPs in our sample, researchers and practitioners should further examine the meaningful health risks and protective effects associated with IVPs.

## Introduction

Intravaginal hygiene practices (IVPs) are often reported in communities with high rates of cervical cancer, including among Haitian women [1]. IVPs usually involve the vaginal insertion of plants, herbs, chemicals, and other agents for various indications including to cleanse, dry, and tighten the vagina; prevent and treat vaginal infections; reduce vaginal odor; and to enhance sexual pleasure [1]. These practices also involve the ingestion of herbal concoctions along with vaginal fogging or steaming [2]. Among Haitian women, IVPs are commonly used for gynecological health maintenance, with products, frequency of use, preparation, and motivations for use largely varying [1,3]. In the United States (U.S.) the burden of cervical cancer is exceptionally high among Haitian migrant women, with the incidence rate being four times higher than the general population [4,5]. While this heightened risk of cervical cancer is attributable to a combination of structural, social, and health-systems factors resulting in delayed detection and treatment, the role of culturally driven health behaviors which can influence disease risk – such as IVPs – remain poorly understood.

Existing evidence suggests a possible link between IVPs and an increased risk of human papillomavirus (HPV) infection, progression, and the development of cervical abnormalities including cervical precancer and cancer [6]. This relationship may be mediated by vaginal dysbiosis, in which earlier studies have linked IVPs to an increased risk of vaginal dysbiosis due to the promotion of epithelial damage and inflammation, reduced protective cervicovaginal mucous secretions, and a reduction of *Lactobacillus* species prevalence [3,7–11]. This disruption to the vaginal environment subsequently enhances vulnerability to sexually transmitted infections (STIs), including HPV which is the primary cause of cervical cancer [7,12,13].

The few studies that have investigated IVPs among Haitian women have not fully explored the broader implications of these practices as they relate to cultural and social contexts, and do not readily account for women’s perspectives regarding this commonly practiced health behavior [11]. Additionally, while certain studies have investigated the clinical outcomes associated with IVPs, research has understudied how IVPs are used within the Haitian community particularly regarding IVP products, motivations, and temporality of use [11]. Understanding cervical cancer risk among Haitian women requires for researchers to go beyond structural determinants and examine culturally rooted health practices that may influence health outcomes.

Among a sample of Haitian migrant women in the U.S., our exploratory study evaluated the following aims: 1) To understand cultural and societal norms shaping Haitian migrant women’s preferences and motivations for engaging in IVPs 2) To identify the gynecologic indications underpinning IVP utilization and characterize IVP products used across these indications. We theorize that this study can aid in developing a heightened understanding regarding IVPs commonly used by women in the Haitian community, and can provide evidence for future research to explore how such practices may influence gynecologic health outcomes including HPV infection and cervical cancer risk.

## Methods

### Ethics statement

Ethical approval for this study was obtained from the Johns Hopkins Medicine Institutional Review Board (IRB00336334). Due to literacy levels largely varying in the Haitian community, oral informed consent was approved by the study IRB [14]. After explaining the purpose of the study to participants, the primary investigator obtained oral consent and completed a waiver of documentation of consent form per the study IRB protocol.

### Theoretical framework

Our study was guided by the PEN-3 Cultural Model (Fig 1). The PEN-3 Model situates culture at the center of determinants of health behavior in health promotion and disease prevention, and endorses the assessment of cultural communities experiencing a health problem to assess culturally unique beliefs, assets, and practices that positively or negatively impact health [15]. The PEN-3 Model is comprised of the following three domains: Cultural identity, Relationships and expectations, and Cultural empowerment. These domains were used to explore cultural and social determinants that could influence IVP engagement among Haitian migrant women [16]. The PEN-3 Model domains informed the development and framing of qualitative interview questions in which participants were asked to describe how social and cultural contexts influenced women’s decision to engage in IVPs and the underlying expectations to perform these practices (Cultural Identity); societal perceptions regarding IVPs including how practices were taught and passed down intergenerationally, and how partner preferences influenced IVPs (Relationships and Expectations); alongside positive and negative experiences and receptions towards IVPs (Cultural Empowerment).

**Fig 1:**
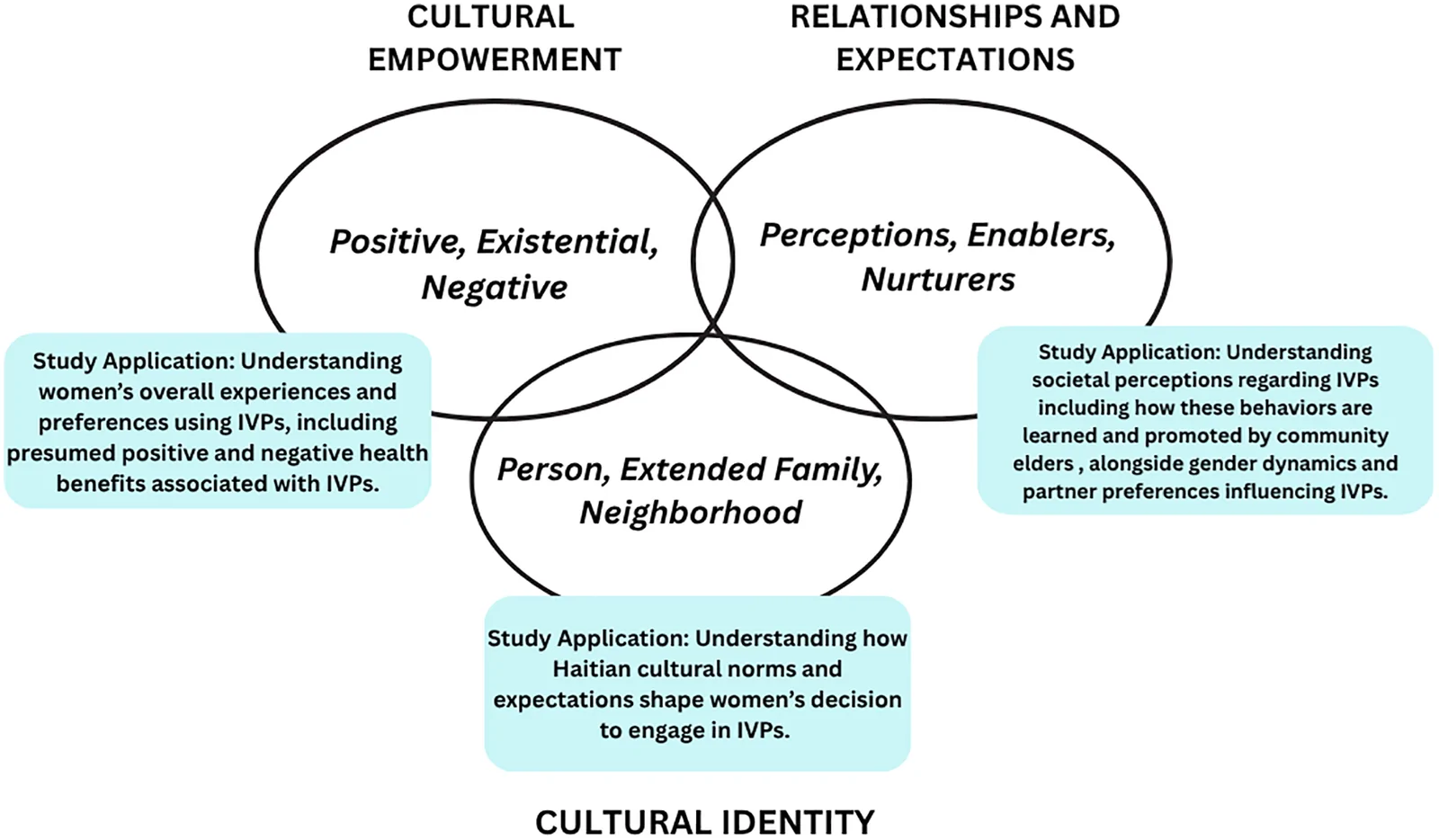
Application of the PEN-3 Cultural Model to the study.

### Study design

Data presented in this manuscript is derived from a larger study exploring factors influencing cervical cancer risk and prevention behaviors among Haitian migrant women [17]. The current manuscript analyzes a subset of data derived from the larger study. Given that this area of research remains understudied, an exploratory sequential mixed-methods design was used as it allows for the exploration of a concept through qualitative data collection followed by quantitative validation [18,19]. This method allows for understanding the complexity and depth of the research phenomenon, while enabling a higher degree of validity and accuracy compared to a single methodological approach [18,19].

Following an exploratory sequential mixed-methods design, the study was conducted in three separate phases (Fig 2): *Phase 1 (qualitative interviews)* involved collecting data through semi-structured in-depth interviews with participants. *Phase 2 (item development and survey administration)* involved the development of items using data derived from qualitative interviews. Items assessed traditional health practices and cultural beliefs influencing women’s cervical cancer risk and behaviors. These items were subsequently psychometrically validated and formed the Traditional Health Practices for Cervical Cancer Prevention Scale (THP-CCP)*.* The development and psychometric validation of the THP-CCP scale are described in a separate manuscript, in which the full scale is provided [20]. Participants who endorsed engaging in IVPs after completing the THP-CCP scale were then asked additional follow-up questions to identify the specific products used and motivations or indications for use (e.g., gynecologic concerns, infection prevention and treatment, menstrual-related cleansing, etc). *Phase 3 (data integration)* involved the integration of qualitative and quantitative data which was done through data reduction and correlating and comparing data. Data were synthesized to identify areas of correspondence and discrepancies, which were integrated within the results [21].

**Fig 2:**
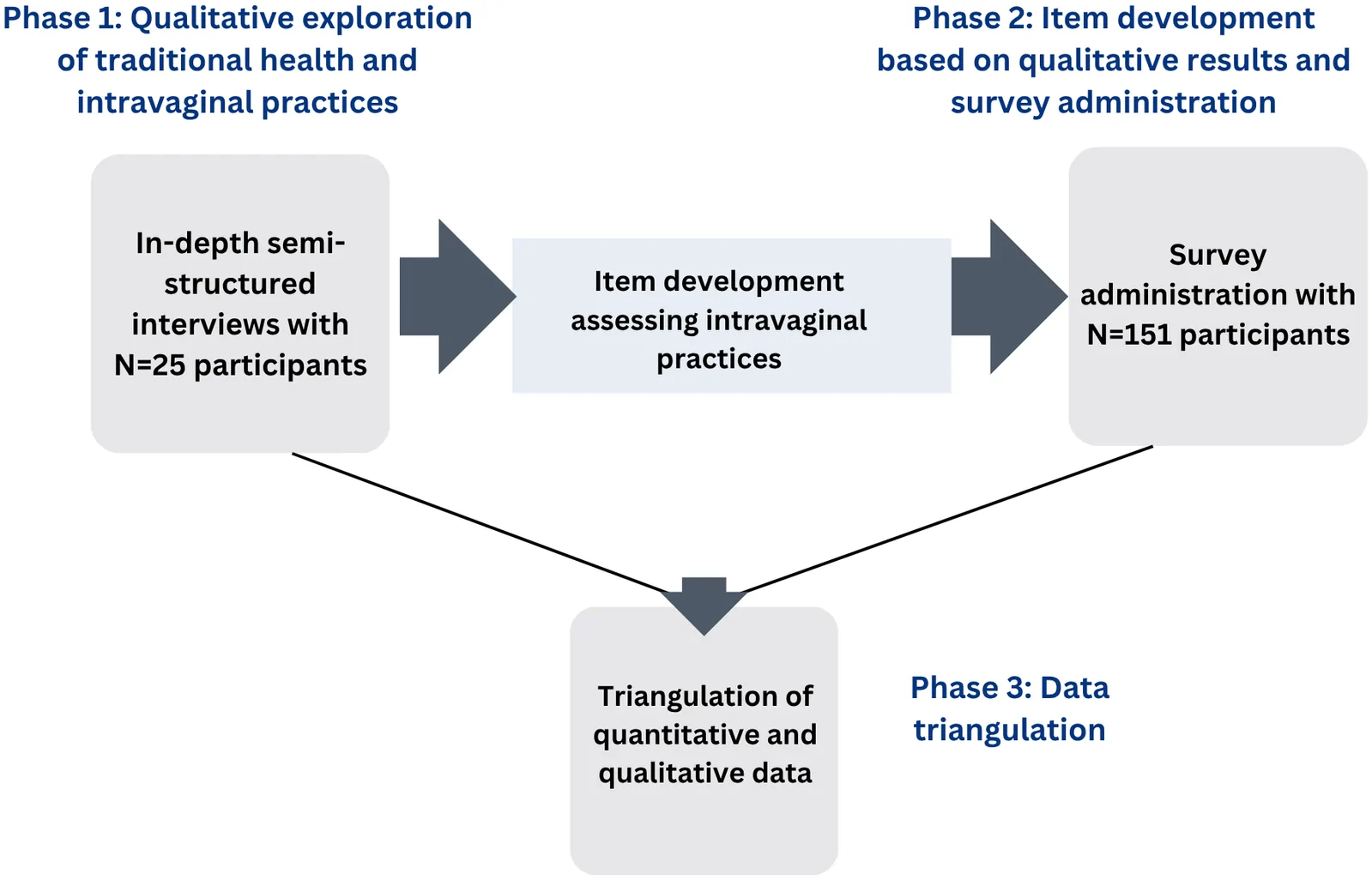
Exploratory sequential mixed-methods study design.

### Setting and eligibility criteria

Women had to meet the following eligibility criteria for participation in the study: a) identify as female b) 18–45 years old c) born in Haiti and residing in South Florida at the time of the study d) not having undergone any procedure involving the removal of the cervix e) history of being sexually active with men f) fluent in Haitian Creole.

Participants were recruited from two community-based centers in South Florida serving the health and social needs of the local Haitian community. South Florida was selected as the geographical area for recruitment as it has the largest Haitian immigrant and migrant population in the U.S.; with Haitian women in this region experiencing cervical cancer incidence rates reaching approximately 38 per 100,000 [14,22] Recruitment took place between May 5^th^ 2023 and was completed September 22^nd^ 2024. All study aims and procedures were explained to participants in Haitian Creole by the research team. Given that the Haitian community has been largely underrepresented in research studies, coupled with the fact that most individuals at recruitment sites were newly arrived migrants, the primary investigator not only explained why the research study was being conducted, but also described how the findings could potentially benefit the Haitian community. This approach appeared to increase interest in study participation and promote trust among participants resulting in a high response rate. Active recruitment strategies were used in which participants were approached by the research team in-person. Passive recruitment was also conducted through flyer posting and distribution. In addition, participants were also asked to share information regarding the study to individuals in their peer network. Due to trust and rapport being established throughout the recruitment process, many participants were proactive in sharing information about the study with their peers. Eligible participants were provided a research incentive of $30 USD for interview completion and $20 USD for survey completion.

### Data collection and analysis procedures

#### Qualitative data collection and analysis.

Qualitative data collection and analysis are detailed elsewhere and are briefly mentioned here [17]. Semi-structured in-depth interviews with n = 25 Haitian migrant women were conducted from May-July 2023. Interview guides were developed from a review of the literature assessing cultural practices influencing cervical cancer risk among Haitian women; along with PEN-3 Model domains [1,4,23–26]. Interview guides were developed in Haitian Creole and contained questions on the following domains: products used for IVPs, indications for IVPs, methods of preparation and frequency of use, and accessibility of products used for IVPs. Translated interview guides are provided in S1 File.

Interviews were conducted in Haitian Creole by the primary investigator who had previous training in qualitative methods. All interviews were audio recorded. Transcripts were kept in Haitian Creole to preserve the depth of participants’ responses [27]. Atlas.ti software was used for qualitative analysis procedures by two coders (DG, AA). Qualitative descriptive analysis methodology with an emphasis on naturalistic and interpretative inquiry was used, with this approach allowing for straightforward descriptions of the research topic under investigation [28]. Open codes were generated and were abstracted into broader themes. A codebook was developed iteratively following MacQueen Codebook Development Guidelines. Two members of the research team (DG, AA) met biweekly to engage in consensus coding in which consistencies and discrepancies amongst the codes were discussed until consensus was reached on the final coding structure [29]. Given that two skilled researchers examined the data, this allowed for researcher triangulation and enhanced the credibility and trustworthiness of the qualitative results [30]. For the purpose of this manuscript, we highlight qualitative findings from the theme pertaining to traditional medicinal practices, which primarily consisted of IVPs. More specifically, we highlight reported IVP preferences, product-types, and indications for use. Interview quotes that are presented in this manuscript were translated from Haitian Creole to English for dissemination purposes.

#### Quantitative data collection and analysis.

Qualitative data informed the quantitative phase, in which data elicited from interviews were used to guide item development for cross-sectional surveys. Survey items evaluated history of engaging in IVPs (measured as a binary variable); products used for IVPs (measured categorically with multiple responses: plant and herbal agents, astringent washes, antibiotics); along with IVP indications (measured categorically with multiple responses: post-partum use, preventing and treating vaginal infections, post-menstrual use, vaginal tightness and dryness, enhancing men’s sexual pleasure). Regarding products used for IVPs, several plant and herbal agents had multiple names in Haitian Creole. Therefore, two individuals from the research team (DG, LA) confirmed the scientific name of each plant and herbal agent with an agronomist based in Haiti.

Open responses were also obtained from participants at the end of the survey to capture additional context regarding IVP practices,and were subsequently analyzed using qualitative analysis procedures (S1 Table). Due to varying literacy levels in the Haitian community, all surveys were interviewer-administered in Haitian Creole. Surveys were conducted in-person at recruitment sites and virtually using HIPPA-compliant Zoom software, depending on participant preference.

Stata 16.1 software was used for quantitative data analysis procedures [31]. Participant baseline characteristics were summarized with counts and frequencies. Chi-square analysis was used to test associations between IVP products and indication categories. Given that we interpreted product-usage within each indication and conducted multiple comparisons, we calculated a Bonferroni adjustment to control for multiple comparisons and to control for type I error. Accordingly, after the Bonferroni adjustment, p-values ≤ 0.004 were considered statistically significant for each individual test.

## Results

The Results are presented in three sections. First, we describe the sociodemographic characteristics of the sample, followed by highlighting preferences for IVPs and the products used for IVPs. Finally, we describe the indications for intravaginal practices.

### Sample characteristics

A total of N = 176 were recruited for this study. Twenty-five (n = 25) women were recruited for the qualitative phase, followed by n = 151 women for the quantitative phase. The mean age of the total sample was 31.8 years. The qualitative sample had a mean age of 34.4 years (SD ± 6.17), while the quantitative sample had a mean age of 31.3 years (SD ± 0.59). Across the total sample, women were primarily from the Northern Department of Haiti, with the majority having migrated to the U.S. less than 1 year prior to participating in the study (n = 121, 72.3%). Additional sociodemographic characteristics of the sample are described in Table 1. Of note, only a subset of sociodemographic information was collected during the qualitative phase, as the primary focus was on eliciting experiential narratives.

**Table 1:** Sample characteristics.

Participant Characteristics	Qualitative Sample N = 25 n(%)	Quantitative Sample N = 151 n(%)
*Age*		
18-24	2 (8.0)	37 (24.5)
26-32	11 (44.0)	48 (31.8)
33-45	12 (48.0)	66 (43.7)
*Origin in Haiti*		
Northern Haiti	8 (32.0)	71 (47.0)
Southern Haiti	5(20.0)	12 (8.0)
Central Haiti	5 (20.0)	19 (12.6)
Western Haiti	6 (24.0)	49 (32.5)
*Length of time in the U.S.*		
Less than 1 year	12 (48.0)	116 (77.9)
Over 1 year	13 (52.0)	33 (22.2)
*Religious Affiliation*		
Catholic	7 (28.0)	43 (29.1)
Christian protestant (Baptist, Methodist, Seventh day Adventist, Lutheran, Nondenominational)	18 (72.0)	105 (80.0)
*Marital Status*		
Single/Divorced/Widowed	3 (12.0)	33 (21.9)
Married	10 (40.0)	36 (23.8)
Single, with partner	12 (48.0)	82 (54.3)
*Highest education level completed*		
Did not graduate high school	--	42 (27.8)
Graduated high school	--	62 (41.1)
More than high school	--	47 (31.1)
*Number of lifetime sexual partners*		
Less than 3 partners	--	95 (62.9)
More than 3 partners	--	18 (11.9)
Prefer not to respond	--	38 (25.2)
*Number of children*		
None	--	74 (49.0)
1-2	--	62 (41.1)
3 or more	--	15 (9.9)
*History of Pap Smear*		
No	--	78 (51.7)
Yes	--	73 (48.3)

### Preferences for intravaginal practices

All participants in qualitative interviews (n = 25, 100%) endorsed IVPs for their gynecologic health. In the quantitative sample, IVPs were endorsed by nearly seventy percent of participants (n = 104, 68.9%). Women detailed how in Haiti, IVPs were frequently used and referred to as either *twalet deba, bain, or vapè,* depending on the preparation, context, and indication for use. Women typically started these practices either during puberty or after their sexual debut. These practices were oftentimes preferred for the prevention and treatment of gynecologic ailments as opposed to going to a Western doctor. This preference was largely due to cultural influences, but also economic constraints. Women described that not having the financial means to pay for a physician consult resulted in many women relying on IVPs. All women in qualitative interviews described learning traditional medicinal practices- including IVPs- from their elders. Women confirmed maintaining these practices upon migrating to the U.S. and described the ease of continuing such practices as South Florida had a large Haitian community which facilitated access to IVP products and supplies.

*“For traditional medicine, this is something we grow up with in Haiti. From a young age, we have our grandmothers who are always explaining these things to us. They always tell us when we finish having our period, we need to use these practices…every parent in Haiti especially if they are raising young women are always boiling different plants… this is how we are raised. This is how we grow up. So as adults we know what to use. If we have a vaginal infection we know which plants to use…if we have a stomachache, we know which plants to use.” – 33 year-old from Au Cayes; migrated less than 1 year ago*

### Products used for intravaginal practices

Women in qualitative interviews described using a variety of IVP products. Products were classified as plants and herbal agents, antiseptic and astringent washes, and antibiotics. The products identified during qualitative interviews were endorsed by women who completed surveys (Table 2).

**Table 2:** Products used for intravaginal practices.

Any IVP use (n = 104, 68.9%)	
IVP Products	n (%)
***Plant and Herbal Agents***	
Ti bonm (Peppermint)	69 (66.3)
Palma christi (Castor leaves)	20 (19.2)
Pwa kongo (Pigeon pea)	74 (71.2)
Fey asosi (Bitter melon)	24 (23.1)
Fey twompet (Trumpet leaves)	41 (39.4)
Jiwòf (Cloves)	19 (18.3)
Other	72 (69.2)
***Antibiotics***	
Metwodinazole and Amox (Metronidazole and Amoxicillin)	61 (58.7)
***Astringent Washes***	
Dlo Twalet (Borasol, Lemisol, Hygisol)	48 (46.2)
Betadine, Pèmaganant (Povidone-iodine, Potassium permanganate)	16 (15.4)
Vineg, Citron (Vinegar, Lime Juice)	15 (14.4)

A diversity of plant and herbal agents were used among participants, with some being commonly known and others being less documented; thus, highlighting the breadth of these agents (Table 2). Over half of participants (n = 72, 69.2%) also endorsed using plant and herbal agents that were not among the predefined survey options. These participants were asked to specify the names of these products which are detailed in S2 Table.

Antiseptic and astringent agents consisted of items such as *dlo twalet* (e.g., boric acid, lactic acid), *betadine* (e.g., povidone-iodine*)*, *vineg* (e.g., vinegar) with 76.0% (n = 79) confirming use of these agents. Antibiotic use was reported by 58.7% (n = 61) of participants in which women described obtaining antibiotics such as *metwonidazole* (metronidazole) and *amox* (amoxicillin) without a prescription from local Haitian vendors.

*“In Florida it is like Haiti. They have vendors to sell everything ok?! They have a ‘machan fey’ that sells all types of things! All the medication that you can find in a pharmacy they sell all those pills, everything you need. You can find people who tell you ‘ Oh here is what you need to treat this thing!’...it is like Haiti… and things are evolving, you can find these products online...’- 43-year-old from Port-au-Prince; migrated 2 years ago*

### Indications for intravaginal practices

IVP indications were identified during qualitative interviews, in which participants described usage for the prevention and treatment of vaginal infections, post-menses use, post-partum use, and for enhancing vaginal dryness and men’s pleasure during sex. These themes were incorporated within the survey, and participants were asked to select all applicable indications for their personal IVP use. Post-menses use was the most commonly reported indication (n = 69, 66.3%), followed by for the prevention and treatment of vaginal infections (n = 55, 52.9%), post-partum use (n = 35, 33.7%), and enhancing vaginal dryness and men’s sexual pleasure (n = 17,16.3%) (Table 3).

**Table 3:** Chi-square analysis of products used for intravaginal practices by indication.

	Indication: Prevention and Treatment of Vaginal Infections (n = 55, 52.9%)		Indication: During or After Menses (n = 69, 66.3%)		Indication: After Pregnancy (n = 35, 33.7%)		Indication: Enhance Vaginal Dryness (n = 17, 16.3%)	
Product	n (%)	p-value	n (%)	p-value	n (%)	p-value	n (%)	p-value
**Plant and Herbal Agents**								
Ti bonm (Peppermint)	46 (83.6)	**p = 0.000**	60 (87.0)	**p = 0.000**	27 (77.1)	**p = 0.002**	14 (82.4)	p = 0.014
Palma christi (Castor leaves)	9 (16.4)	p = 0.392	11 (15.9)	p = 0.370	14 (40.0)	**p = 0.000**	5 (29.4)	p = 0.037
Pwa kongo (Pigeon pea)	41 (74.5)	**p = 0.000**	49 (71.0)	**p = 0.000**	24 (68.6)	p = 0.008	17 (100.0)	**p = 0.000**
Asosi (Bitter melon)	13 (23.6)	p = 0.049	15 (21.7)	p = 0.072	11 (31.4)	**p = 0.004**	6 (35.3)	p = 0.020
Fey twompet (Trumpet leaves)	26 (47.3)	**p = 0.000**	29 (42.0)	**p = 0.000**	9 (25.7)	p = 0.823	6 (35.3)	p = 0.423
Jiwòf (Cloves)	12 (21.8)	p = 0.010	13 (18.8)	p = 0.033	2 (5.7)	p = 0.162	5 (29.4)	p = 0.026
Other	43 (78.2)	**p = 0.000**	47 (68.1)	**p = 0.000**	19 (54.2)	p = 0.372	9 (52.9)	p = 0.645
**Antibiotics**								
Metwonidazole and Amox (Metronidazole and Amoxicillin)	33 (60.0)	**p = 0.000**	35 (50.7)	p = 0.018	21(60.0)	p = 0.007	8 (47.1)	p = 0.552
**Astringent Washes**								
Dlo Twalet (Borasol, Lemisol, Hygisol)	23 (41.8)	p = 0.045	27 (39.1)	p = 0.076	17(48.6)	p = 0.015	8 (47.1)	p = 0.151
Betadine/Pèmeganat (Povidone-iodine, Potassium permanganate)	16 (29.1)	p = 0.081	11 (15.9)	p = 0.050	5 (14.3)	p = 0.418	4 (23.6)	p = 0.066
Vineg/Citron (Vinegar, Lime Juice)	8 (14.6)	p = 0.152	11 (15.9)	p = 0.024	3 (8.00)	p = 0.759	3 (17.6)	p = 0.259

*Bolded results indicate values demonstrating significance using the Bonferroni-adjusted threshold (p ≤ 0.004).

#### Prevention and treatment of vaginal infections.

The preparation and use of specific plants and herbal agents for the prevention and treatment of vaginal infections was well-described during qualitative interviews. Women often prepared plants and herbal agents as teas for oral intake and for intravaginal application as this was viewed as the most effective approach for preventing and treating vaginal infections. Women highlighted that the effectiveness of these agents largely depended on how they were prepared, with preparation methods varying considerably. Certain women believed that the ingestion of teas aided with systemic cleansing and treatment, while direct intravaginal use aided with symptomatic management (S1 Table). For instance, some women boiled multiple plants together and drank the end-product as a tea, and would wait until the remaining liquid was cooled for intravaginal application. Other participants described that hot water should instead be poured over the plants, as boiling them directly reduced the efficacy. Plant and herbal agents associated with the prevention and treatment of vaginal infections in the chi-square analysis included *ti bonm* (peppermint; χ^2^ = 29.99, p ≤ 0.001), *pwa kongo* (pigeon pea; χ^2^ = 22.59, p ≤ 0.001), *fey twompet* (trumpet leaves;χ^2^ = 17.71, p ≤ 0.001) and *other agents* (χ^2^ = 32.26, p ≤ 0.001), which corresponded with qualitative reports describing their use and perceived efficacy for the prevention and treatment of vaginal infections.

*“Untreated water [in Haiti] used to give me infections. I would go to the country-side, and I would wash vaginally with the untreated water, and I would feel [vaginal] itching, itching, itching! The hospital would ask for too much money, so I would use plants. I would boil the plants [fey twompet and ti bonm] and I would drink it, and use the rest vaginally. After 3 days, I no longer would have any symptoms.” –34-year-old from Port-au-Prince; migrated less than 1 year ago*

Similar to other IVP products, participants reported it was common practice to purchase oral antibiotics without a prescription through street vendors. Women were also able to do this through local Haitian botanicas. As a result, when women experienced vaginal symptoms, many would purchase antibiotics from local vendors as opposed to presenting to a health center.

*“Women purchase pills and think they are purchasing antibiotics but actually are purchasing other medications without them knowing. For instance, you have an infection and you’re taking a pill for several days and you think you’re taking amoxicillin, but it happens to be something else that they gave you. Women insert medications vaginally, and they drink whatever it is that they purchase…” – 33-year-old from Fort Liberte; migrated less than 1 year ago*

Antibiotics were associated with the prevention and treatment of vaginal infections in the chi-square analysis (χ^2^ = 12.81, p ≤ 0.001). While antiseptic and astringent washes did not demonstrate statistical significance in the chi-square analysis, the use of these products for preventing and treating vaginal infections were well-described in qualitative interviews.

*“Women buy borasol (boric acid) at times when they need relief [from their symptoms]. They put it inside them to treat infections. When someone has a vaginal infection that is unrelieved, I usually hear that women specifically use vinegar for the infection. I knew a young lady who had an infection that was causing really bad symptoms. She went to the doctor [in Haiti] and they gave her vinegar for her to do twalet deba (vaginal cleansing). She kept using it until her symptoms went away.” – 41-year-old from Anse a Veau; migrated less than 1 year ago*

#### Postpartum use.

The postpartum period was a common indication for IVPs, in which women described how IVPs during the postpartum period was a cultural expectation. Of the n = 73 women who had children in surveys, nearly half (n = 35, 48.0%) endorsed IVPs during the postpartum period. The preparation for use during the post-partum period differed from that of vaginal infections. More specifically, a *bain or vapè* was concocted which involved boiling several plants and herbal agents and letting the steam enter vaginally. This was believed to promote healing, removal of excess fluids and blood, and to enhance vaginal tightening. Women also drank teas prepared with plants and herbal agents as this was believed to help prevent infections after giving birth. It was described how vaginal tightening during the postpartum period was critical as this was also the preference of male partners.

*“There are a lot of people when they are doing health related practices they believe more in teas and natural medicine. In our culture, when a woman has a baby it is not only, ‘ok yea you go to the doctor, you deliver, you take antibiotics and you’re done.’ In our culture, even when you leave the hospital after 2 or 3 days you see our mothers looking for plants, preparing vapè, they do all of those things…it is already in our mentality, even when we have children in the United States we have to do bain (baths), drink teas, do all of those things.” – 30-year-old from Port-au-Prince; migrated 2 years ago*

The frequency and duration of these practices varied, with some women engaging in IVPs for approximately two weeks after delivery, while other women described using these practices for several months. Chi-square analysis revealed statistically significant associations between postpartum use and *ti bonm* (peppermint; χ^2^ = 9.58, p = 0.002), *palma christi* (castor leaves; χ^2^ = 28.58, p ≤ 0.001), and *asosi* (bitter melon; χ^2^ = 8.22, p = 0.004).

#### Use following menstruation.

IVPs after one’s menstrual cycle were well-described during qualitative interviews, and a substantial number of women endorsed this practice on surveys (n = 69, 66.3%). In qualitative interviews, women voiced that there was a notion that menstruation was a period where women were not ‘clean’, and as a result women undertook further action to enhance vaginal cleanliness.

*“When I feel like I have a different odor in my urine and every time I finish my cycle I always boil ti bonm (peppermint)* with *pwa kongo (pigeon pea) and drink it with ampicillin and amoxicillin which are antibiotics…when we drink the antibiotics with tea it relieves you and you see a difference in your urine.” 33-year-old from Au Cayes; migrated less than 1 year ago*

Several plants and herbal agents were associated with use after menses including *ti bonm* (peppermint; χ^2^ = 54.6, p ≤ 0.001), *pwa kongo* (pigeon pea; χ^2^ = 24.62, p ≤ 0.001), and *fey twompet* (trumpet leaves; χ^2^ = 14.22, p ≤ 0.001). Additionally, it was common for women to take a one or two-week course of antibiotics after their menstrual cycle due to beliefs that this had additional cleansing properties. Survey data revealed that among women who used antibiotics, 86.3% (n = 44) reported use after one’s menstrual cycle with this being the most common indication for antibiotic use. Oftentimes, women combined plant and herbal agents with antibiotics as this was believed to have an enhanced effect. While antibiotic use reached conventional statistical significance in the chi-square analysis, it did not retain significance after applying the Bonferroni correction.

#### Vaginal dryness and men’s pleasure.

Using IVPs to enhance vaginal dryness and men’s pleasure was well-described in qualitative interviews. Interestingly however, very few women directly confirmed engaging in these practices and instead provided extensive examples of peers who engaged in such practices. This was also seen in our survey, in which a small number of women (n = 17, 16.3%) confirmed engaging in IVPs for enhancing vaginal dryness and men’s pleasure.

*“Ti bonm (peppermint), it helps a lot. For example, when you have a baby, you can take antibiotics, but you also use teas made with plants to dry your insides…to make your insides very tight and very firm….men prefer when women keep their insides healthy and firm.” – 34-year-old from Port-au-Prince; migrated less than 1 year ago*

Women voiced that in Haiti, there was a societal expectation of maintaining vaginal dryness which was rooted in the preferences of men; and that the presence of vaginal fluid was associated with infections. Several women claimed this mentality was largely due to insufficient sexual and reproductive health education in Haiti resulting in widespread misconceptions regarding the female body. Many women voiced that even as children they were told by female elders that it was important to use certain products to maintain vaginal dryness as this is what men preferred.

*“There is a level of criticism that women receive that make them do anything, women will do anything! They will drink teas, use washes, women do this because of men. The men won’t accept that a woman has vaginal liquid that is supposed to protect her vagina. Instead, there are men who see this, and they say that the women’s vagina isn’t good, that it isn’t clean!”- 33-year-old woman from Fort Liberte; migrated less than 1 year ago*

Among plant and herbal agents, *pwa kongo* was associated with use for vaginal dryness and men’s pleasure (pigeon pea; χ^2^ = 19.93, p ≤ 0.001). Women in qualitative interviews detailed how astringent washes were commonly used for men’s pleasure however this did not reach statistical significance after applying the Bonferroni correction.

## Discussion

Our mixed-methods study utilized a cultural framework to explore IVP preferences, products, and indications for use among Haitian women who have recently migrated to the U.S. While numerous studies suggest that IVPs may contribute to the risk of gynecologic conditions including HPV infection and progression, few studies have comprehensively explored motivations for IVP use within the Haitian context [32]. Similar to studies conducted in other settings, our findings demonstrated that women had varied motivations for IVPs which were partly driven by adherence to social and cultural norms surrounding womanhood particularly in relation to their sexual and reproductive health [33,34].

IVPs among Haitian women in our sample included a combination of products and modalities for use in which women both drank oral preparations and used agents intravaginally. The combination of multiple product types (e.g., plant and non-plant agents) was common among women in our study, in addition to women describing the use of ingesting agents orally coupled with intravaginal application. This aligns with research conducted by Hilber et al. (2012) in which it was reported that women in sub-Saharan Africa and Southeast Asia were found to engage in vaginal steaming and cleansing along with oral ingestion with intended effect on the vagina or uterus [2,26]. Yet, the literature assessing IVPs among Haitian women has solely focused on intravaginal application of a limited number of agents while not accounting for oral ingestion or combining multiple products, including non-prescribed antibiotics.

Plant and herbal agents were commonly used for IVPs. Interestingly, *pwa kongo* (pigeon pea) was the most frequently used plant agent and was strongly associated with nearly all IVP indications; with it being the most frequently used agent for enhancing vaginal dryness and men’s sexual pleasure. Seay et al. (2017) reported that *pwa kongo* was commonly used in a sexual context in Haiti and was associated with an increased odds of high-risk HPV infection among women [1]. Given these findings, it was suggested that certain intravaginal agents may contribute to heightened risk of vaginal dysbiosis and HPV infection among Haitian women [1]. However, it is also important to consider whether such agents and practices may harbor some benefit. For instance, *ti peta* (Guinea henweed) which was used by a small percentage of participants, has been reported to be widely used in traditional medicine across several countries with certain studies demonstrating its ability to prevent certain infections, reduce inflammation, and potentially regulate cancer cell growth [35]. The net effects- both harmful and beneficial- of using these products needs to be further explored.

Nearly 60% of participants endorsed the use of unprescribed antibiotics. High rates of antibiotic use in this sample are of concern, given that this may contribute to antimicrobial resistance and changes in the vaginal microbiota [36]. In qualitative and quantitative arms, it was found that women commonly used antibiotics after their menstrual cycle. Such frequent use of unprescribed antibiotics has been found to contribute to recurrent bacterial vaginosis; thus, increasing the risk of HPV acquisition and other gynecologic ailments [37]. Similar concerns exist for the frequent use of astringent washes which was used by nearly eighty percent of women in our sample.

It was well-described in qualitative interviews how there was societal pressure for vaginal dryness and tightness, with this pressure being derived primarily from men and from elders. As a result, women used certain agents to enhance desirability. In the qualitative sample, all women voiced learning about IVPs and the importance of vaginal dryness in childhood, and began using IVPs either after puberty or sexual debut. While many women denied the direct use IVPs specifically for vaginal tightness and men’s pleasure, numerous women voiced that during the postpartum period IVPs were critical for vaginal tightness, drying, and the removal of excess fluid which was viewed as undesirable. Global guidelines are limited for perineal, vulvar, and vaginal hygiene and when to resume sexual activity after birth and lack consensus [38]. In addition, evidence related to best practices for hygiene after birth is limited [38]. The high rates of IVPs reported during the postpartum period, highlights the need for more research to generate more evidence on best practices. Previous studies in sub-Saharan Africa have reported that IVPs are rooted in cultural norms and gendered expectations in which the use of certain natural products are an expectation that women are expected to fulfill, with vaginal wetness being associated with infection, moral uncleanliness, and promiscuity [32–34]. Our results yielded similar findings, in which women stated that in Haitian culture vaginal fluid was viewed by men and society at-large as unclean and was associated with infections. This resulted in a preference for vaginal dryness which was also endorsed by community elders. Given this finding, future research should also consider whether IVP use after pregnancy serves as a proxy for enhancing vaginal tightness among Haitian women.

### Limitations

This study was not without limitations. There was a potential for social desirability bias during data collection procedures. However, this was mitigated by ensuring participant anonymity and triangulation of qualitative and quantitative data. Although several product-indication associations were significant at the standard p < 0.05 level, they did not withstand correction for multiple comparisons using the Bonferroni method. It is possible that associations that did not demonstrate significance after correction may still reflect meaningful patterns and should be further explored in studies with larger sample sizes. Lastly, given the sampling methods used, our results may not apply to all Haitian women. IVPs may significantly vary between individuals from urban versus rural areas and thus should be taken into account when interpreting results.

### Future research

Clinicians working with Haitian women and other diverse populations need to be aware of the commonality of IVPs when addressing health behaviors. While traditional medicine is widely used globally, there is a limited understanding of how these practices are used across different settings and populations [20,39]. The results from this study can help clinicians identify key timepoints in which women are more likely to use IVPs, and therefore can aid clinicians in providing appropriate counseling and guidance on IVP use and potential health risks. The current literature on IVPs primarily focuses on the adverse effects associated with this practice, yet it could be possible that certain IVP practices – particularly the use of specific agents– may confer certain health benefits, however further research is needed to determine this [20]. Clinicians should be cautious in framing these practices negatively as IVPs have been well-trusted and used intergenerationally within the Haitian community. Therefore, future research should explore diverse approaches in bridging traditional and western medicinal practices and beliefs in a culturally responsive manner. Of note, the quantification of IVP exposure on disease risk is especially challenging as there are numerous practice characteristics that shape IVPs; specifically, the use of various agents at various frequencies, methods of application, timing with sex, different indications with patterns of use,and IVP products differing considerably across regions [1,2,40]. Research with robust designs should be conducted to explore the link between IVP exposure and gynecologic health outcomes [41].

Finally, most women learned about IVPs in childhood with the majority engaging in IVPs either during puberty or after sexual debut and maintained these practices after migration. Future research should also explore whether the age at which IVPs begin, migration status, and level of acculturation, influences future health decision-making.

## Supporting information

S1 File This file contains the interview guide used during qualitative interviews. (DOCX)

S1 Table This file contains open survey responses on IVP products and indications for use. (DOCX)

S2 Table This file contains a table with additional IVP agents that participants endorsed using. (DOCX)
