# Supplementary material for: Understanding intravaginal practices for gynecologic health among Haitian migrant women: A mixed-methods analysis
Source: PLOS Glob Public Health. 2026 Oct 29;6(10):e0007394. doi: 10.1371/journal.pgph.0007394 (PMC13719032; doi:10.1371/journal.pgph.0007394)
Supplement: Supplementary file 1 [file pgph.0007394.s001.docx]

S1 File. **Traditional Health and Intravaginal Practices Interview Questions**

*I would now like to get to know a little bit more about cultural and traditional practices in the Haitian community that influence women’s health in general and their sexual and reproductive health.*

1. In Haiti , where do most women typically get their health care?
   a. Are Haitian women more likely to seek care from physicians? Are they more likely to use traditional medicine?
2. For Haitian women who have migrated to the United States, how do they usually navigate the health care system? Where do they typically get their health care?
   a. Are Haitian women more likely to seek care from physicians? Are they more likely to use traditional medicine?
3. Can you describe how traditional medicine is viewed in the Haitian community?
   a. When might a Haitian woman go both to a western doctor ?
   b. When might a Haitian woman go to a traditional doctor?
   c. When might a Haitian women consult both a western and a traditional doctor at the same time?
4. There are many natural remedies such as plants and herbs that are used for different types of illnesses in the Haitian community. Are you able to describe them for me?
5. There are also different practices that Haitian women use for intimate hygiene and gynecologic health. In Haiti this is usually referred to as twalet deba or vapè. Are you able to describe these practices?
6. Do you use products intravaginally for your gynecologic health? If so, can you describe which products or remedies you use ?
   a. What products are used intravaginally?
   b. What products are used orally?
   c. How do women use these products?
   d. In what situations do you use intravaginal products instead of oral products?
   e. How are these products prepared?
   f. How frequently are they used?
   g. Where are you able to purchase these products (in Haiti and in the United States)?
   h. Can you describe how you were taught you how to use them? Who taught you how to use them?
7. For what reasons do Haitian women use different products for their gynecologic health? For instance, to treat vaginal infections, to prevent vaginal odor, vaginal cleansing, etc.?
   a. Does the preparation of these products differ depending on the use/indication? If so, can you provide more detail?
8. How important is vaginal cleanliness among Haitian women, and how is it understood?
   a. What does vaginal cleanliness mean to Haitian women?
   b. How do individuals in the community at-large view vaginal health and cleanliness? How do these views differ between men and women?
9. How is gynecologic health viewed among Haitian women?
   a. How do individuals in the community at-large view gynecologic health? How do these views differ between men and women?
10. I’ve heard that women often use intravaginal practices due to male or partner preferences for vaginal dryness. Can you tell me more about this?
11. In many cultures, it is common for elders to educate young girls about vaginal health and cleansing. Are you able to describe this dynamic within the Haitian community?
12. For gynecologic health, are women more inclined to trust traditional medicine as opposed to western medicine?
    a. Can you describe overall preferences for intravaginal practices? How are these practices maintained upon migrating to the United States?
13. What other things that Haitian women do to maintain their gynecologic health?
