# Supplementary material for: Understanding intravaginal practices for gynecologic health among Haitian migrant women: A mixed-methods analysis
Source: PLOS Glob Public Health. 2026 Oct 29;6(10):e0007394. doi: 10.1371/journal.pgph.0007394 (PMC13719032; doi:10.1371/journal.pgph.0007394)
Supplement: Supplementary file 2 [file pgph.0007394.s002.docx]

**S1 Table. Open Responses from Surveys**

| IVP Indications | Qualitative Quote | Participant Characteristics |
| --- | --- | --- |
| Postpartum Use |  |  |
|  | “If you don’t use *bain* (vaginal bath) [after delivery] your vagina won’t be normal. My sister had issues after delivery…an aunt of mine went to look for plants in the wild bushes, she boiled them and made my sister sit on the bucket and her vagina became normal again…my sister decided to stop going to the hospital for care after that.” | 36-year-old from Northern Haiti, migrated less than 1 year ago |
|  | “They say after you deliver you have to use *vapè* (vaginal steaming) because your vagina is no longer tight and it gives an odor.” | 21-year-old from Northern Haiti, migrated less than 1 year ago |
| Post-menses Use |  |  |
|  | “*Fey twompet* (trumpet leaves) you drink it and ti bonm (mint leaves) you boil it after you finish with your period.” | 34-year-old from Western Haiti, migrated less than 1 year ago |
|  | “The nurse in the clinic in Haiti told me that I need to take *amox* (amoxicillin) each time I finish my menstrual cycle.” | 23-year-old from Northern Haiti, migrated less than 1 year ago |
| Preventing and treating vaginal infections |  |  |
|  | “ If you drink tea [instead of using plants vaginally] it can help more with an infection because the illness is on the interior not the exterior [of your body]. ” | 34-year-old from Central Haiti, migrated less than 1 year ago |
| Vaginal Dryness and Men’s Pleasure |  |  |
|  | “Women are using *vapè* (vaginal steaming) for [vaginal] tightening. A lot of times, young girls are also selling products on the street and are asking people to purchase medications- *amox* (amoxicillin) and *borasol* (boric acid) -from them.” | 36-year-old from Southern Haiti, migrated less than 1 year ago |
|  | “Their vaginas become so dry [after steaming]! The elders do this often because they have a lot of children, they may have seven children and they do the steaming in a big bucket. They put the steaming water in the big bucket and they sit on it. It helps them live longer, many live until they are over 100 years old.” | 36-year-old from Northern Haiti, migrated less than 1 year ago |
|  | “They also use vinegar often to make their vaginas even drier; they pour vinegar into the hot and they do their *twalet* (vaginal washing).” | 40-year-old from Western Haiti, migrated less than 1 year ago |
