# Supplementary material for: Understanding intravaginal practices for gynecologic health among Haitian migrant women: A mixed-methods analysis
Source: PLOS Glob Public Health. 2026 Oct 29;6(10):e0007394. doi: 10.1371/journal.pgph.0007394 (PMC13719032; doi:10.1371/journal.pgph.0007394)
Supplement: Supplementary file 3 [file pgph.0007394.s003.docx]

**S2 Table. Other Agents used for Intravaginal Practices**

| Haitian Creole Name(s) | English Name(s) | Scientific Name | Frequency Counts |
| --- | --- | --- | --- |
| Plant and Herbal Agents |  |  |  |
| Zanmoret ,berejèn mawon | Turkey berries, Pea eggplants | *Solanum torvum* | 12 |
| Kre Kakok | Cockscombs | *Celosia* | 20 |
| Ti peta | Guinea henweed | *Petiveria alliacea* | 9 |
| Fey la choy | Sage | *Salvia officinalis* | 2 |
| Koton rouge | Barbados Nut, Purging Nut, Physic Nut | *Jatropha curcas* | 1 |
| Safrann | Turmeric Plant | *Curcuma longa* | 2 |
| Lay | Garlic | *Allium sativum* | 4 |
| Gwayav | Guava | *Psidium guajava* | 12 |
| Papaye | Papaya | *Carica papaya* | 4 |
| Mango | Mango | *Mangifera indica* | 1 |
| Fey zaboka | Avacado leaves | *Persea americana* | 2 |
| Kachiman | Custard apple | *Annona squamosa* | 3 |
| Fey kamomil | Chamomile leaves | Matricaria chamomilla | 3 |
| Bouldimas | Moses-in-the-cradle | *Tradescantia spathacea* | 1 |
| Gingembre | Ginger | *Zingiber officinale* | 4 |
| Persil | Parsely | *Petroselinum crispum* | 3 |
| Kanel | Cinnamon | *Cinnamomum verum* | 5 |
| Semi Kontra | Epazote, wormseed | *Dysphania ambrosioides* | 1 |
| Fey gwo tin | Spanish thyme | *Plectranthus* | 1 |
| Bazilik | Basil leaf | Ocimum basilicum L | 1 |
| Langachat,languichat | Snake plant | *Chromolaena odorata* | 1 |
| Korosol | Soursop plant | *Annona muricata* | 2 |
| Kafe | Coffee leaves | *Coffea arabica* | 1 |
| Kalbas kouran | Calabash leaves | *Crescentia cujete* | 6 |
| Additional Agents |  |  |  |
| Biere | Beer | --- | 2 |

*Of note, certain plant and herbal agents had several names in both Haitian Creole and English, thus for clarification scientific names have been provided.
